# Different Susceptibility to Neurodegeneration of Dorsal and Ventral Hippocampal Dentate Gyrus: A Study with Transgenic Mice Overexpressing GSK3β

**DOI:** 10.1371/journal.pone.0027262

**Published:** 2011-11-03

**Authors:** Almudena Fuster-Matanzo, María Llorens-Martín, Elena Gómez de Barreda, Jesús Ávila, Félix Hernández

**Affiliations:** 1 Centro de Biología Molecular “Severo Ochoa” (CSIC/UAM), Universidad Autónoma de Madrid, Cantoblanco, Madrid, Spain; 2 CIBERNED, Centro de Investigación Biomédica en Red de Enfermedades Neurodegenerativas, Madrid, Spain; New York State Institute for Basic Research, United States of America

## Abstract

Dorsal hippocampal regions are involved in memory and learning processes, while ventral areas are related to emotional and anxiety processes. Hippocampal dependent memory and behaviour alterations do not always come out in neurodegenerative diseases at the same time. In this study we have tested the hypothesis that dorsal and ventral dentate gyrus (DG) regions respond in a different manner to increased glycogen synthase kinase-3β (GSK3β) levels in GSK3β transgenic mice, a genetic model of neurodegeneration. Reactive astrocytosis indicate tissue stress in dorsal DG, while ventral area does not show that marker. These changes occurred with a significant reduction of total cell number and with a significantly higher level of cell death in dorsal area than in ventral one as measured by fractin-positive cells. Biochemistry analysis showed higher levels of phosphorylated GSK3β in those residues that inactivate the enzyme in hippocampal ventral areas compared with dorsal area suggesting that the observed susceptibility is in part due to different GSK3 regulation. Previous studies carried out with this animal model had demonstrated impairment in Morris Water Maze and Object recognition tests point out to dorsal hippocampal atrophy. Here, we show that two tests used to evaluate emotional status, the light–dark box and the novelty suppressed feeding test, suggest that GSK3β mice do not show any anxiety-related disorder. Thus, our results demonstrate that *in vivo* overexpression of GSK3β results in dorsal but not ventral hippocampal DG neurodegeneration and suggest that both areas do not behave in a similar manner in neurodegenerative processes.

## Introduction

GSK3 is a kinase present in most tissues and is particularly abundant in the brain [1]. There are two isoforms of the enzyme termed GSK3α and GSK3β [1]. GSK3 is known to participate in multiple signaling pathways coupled to receptors for a variety of signaling molecules such as insulin or wnt among many others [2]. Aberrantly increased GSK3 activity is believed to play a key role in the pathogenesis of chronic metabolic disorders like type-II diabetes [3], as well as of CNS conditions such as bipolar mood disorder [4], schizophrenia [5], diseases like Huntington's disease [6], frontotemporal dementia with parkinsonism linked to chromosome 17 [7] and Alzheimer disease [8]. With regard to GSK3 and neurodegeneration, increased GSK3 activity has been reported to result in neuronal apoptosis and GSK3 inhibitors have been shown to exert antiapoptotic and neuroprotective effects in many different cell and mouse models [9], [10], [11]. Accordingly, potent and specific GSK3 inhibitors are currently under development [12], [13], [14].

Recent evidences have established that there are differences among dorsal and ventral hippocampal areas, at least in rodent [15]. All these differences are associated with functional specialization, as studies with lesions in dorsal or ventral hippocampus demonstrate [16]. Thus, dorsal regions are involved mainly in memory and learning processes, while ventral areas are related with anxiety, affective or emotional processes [17]. That regionalized processes correlate at genetic and cellular levels, showing that DG is not uniform and that there exist a regionalized specialization [15]. Those studies can be likely translated to human. Thus, the dorsal hippocampus corresponds to the posterior hippocampus in primates, while the ventral correspond to the anterior hippocampus in primates [15].

Here, we have first analyzed GSK3β levels in both DG areas in wild-type mice and explored the effect of GSK3β overexpression in both dorsal DG (dDG) and ventral DG (vDG) in a mouse model with increased GSK3β levels in those hippocampal areas [18]. This animal model exhibits a memory deficit [19], [20] and impaired synaptic plasticity [21]. We demonstrate that ventral hippocampus withstands a neurodegenerative signal as an increase in GSK3β levels better than dorsal hippocampus. In good agreement, evaluation of anxiety-related tests shows a normal behaviour.

## Materials and Methods

### Animals and tissue processing

#### Animal care

Mice were obtained from the Centro de Biologıía Molecular and treated following the guidelines of Council of Europe Convention ETS123, recently revised as indicated in the Directive 86/609/EEC. Animal experiments were performed under protocols (P15/P16/P18/P22) approved by the Centro de Biología Molecular Severo Ochoa Institutional Animal Care and Utilization Committee (CEEA-CBM), Madrid, Spain.

GSK3β mice were generated as described previously [18]. Briefly, GSK3β mice were bred by crossing TetO mice (carrying the bi-direccional tet-responsive promoter followed by the GSK3β and β-galactosidase cDNAs, one in each direction) with CamKIIα-tTA mice. The dual transgenic mice were designated GSK3β, and they overexpress GSK3β in the cortex and hippocampus. Transgenic mice as well as wt mice (C57BL/6) were bred at the *Centro de Biología Molecular “Severo Ochoa”* (Madrid, Spain) and the mice were kept on a normal light-dark cycle (12 hours light/12 hours dark), with free access to food and water.

#### Tissue processing

Animals were killed and the brain was removed, post-fixed overnight in 4% PFA and 30 µm sagittal sections were obtained on a cryostat.

### Volumetric measurement of dentate gyrus atrophy

For volumetric measurement, thionine stained section areas of dentate gyrus were delineated and measured by means of Methamorph image-analysis system. The total volume (mm3) of each granule cell layer was achieved by integration of areas (mm2) with the distance between each sagital plane (mm). The points for integration were 0.36 mm (Fig 104 of the atlas of Paxinos and Franklin [22]) and 3.00 mm (Fig 126 of the same atlas) with respect to the midline. In those sections were dentate gyrus is not divided in two areas, dorsal and ventral areas were measured taking into account the middle point.

### Western blot analysis

Brains were quickly dissected on an ice-cold plate. Hippocampus was isolated and horizontally cut in two equals halves, the dorsal and the ventral hippocampus. Extracts for Western blot analysis were prepared by homogenizing the dorsal and ventral hippocampus from 2 months-old wildtype and transgenic animals in ice-cold extraction buffer consisting of 50 mM Tris HCl, pH 7.4, 150 mM NaCl, 1% NP-40, 1 mM sodium orthovanadate, 1 mM EDTA, a protease inhibitor cocktail (Roche) and 1 µM Okadaic acid (phosphatase inhibitor). The samples were homogenized and protein content was determined by Bradford. Thirty micrograms of total protein were electrophoresed on 10% SDS-polyacrylamide gel and transferred to a nitrocellulose membrane (Schleicher & Schuell, Keene, NH). The experiments were performed using the following primary monoclonal antibodies: anti-GSK3β (1/1000) (BD Transduction Laboratories), anti-p21/9- GSK3α/β (1/500) (Cell Signalling), anti-Akt (1/1000) and anti-phospho-Akt (Ser473) (1/1000) (both from Cell Signalling) and anti-actin (1/5000) (Sigma). The following anti-tau antibodies were used: PHF-1 (1/200, a kind gift from Dr. Davis) reacts with tau when serines 396 and 404 are phosphorylated [23] and 7.51 (1/200; a kind gift from Dr Wischik) which recognizes segments of the last two repeats within the microtubule binding domain of tau in a phosphorylation-independent manner [24]. The filters were incubated with the antibody at 4°C overnight in 5% nonfat dried milk. Secondary goat anti-mouse and anti-rabbit antibodies (1/1000; Invitrogen, San Diego, CA) and ECL detection reagents (Amersham Biosciences, Arlington Heights, IL) were used for immunodetection. Quantification was performed by densitometric scanning. The densitometry values were obtained in the linear range of detection with these antibodies. These values were normalized with respect to the values obtained with an anti-β-tubulin antibody to correct for total protein content.

### Immunostaining

For GFAP immunohistochemistry, sections were rinsed in PBS and incubated in blocking solution (PBS/BSA/FBS/Tx-100) followed by an overnight incubation at 4° with the primary antibody: rabbit anti-GFAP (1/500) (Promega). Then, sections were washed and incubated for 1 hour with the goat anti-rabbit biotinylated antibody (1∶400, Vector) and then another hour with an avidin-biotin-peroxidase complex complex (ABC, 1∶250, Vector). The antibody staining was finally visualised with diaminobenzidine (DAB, 0.05%, Sigma). Images were taken using an Axioskop 2 plus microscope and a CCD camera (Coolsnap FX color).

For immunofluorescence, immunostaining was carried out following a standard procedure. Sections were incubated with the primary antibody overnight at 4°C in a PB solution containing BSA 1% and TritonX-100 1%. The following antibodies were used: mouse anti-Myc, (1/100) (Hibridoma) and rabbit anti-fractin (1/500) (BD Pharmingen). After washing with blocking solution 3 times, sections were incubated with donkey Alexa-conjugated secondary antibodies (anti-rabbit, anti-mouse Alexa-Fluor 488/555/633-conjugated) overnight at 4°C (1∶1,000) (Molecular Probes, Millipore). Finally, after washing with PB solution, sections were incubated with DAPI (1/5,000) (Calbiochem) for 10 minutes.

### Cell counting

Fractin- and reactive GFAP-positive cells were quantified on a series of slices of dorsal and ventral dentate gyrus (DG) from 2 months-old wildtype and transgenic mice using an inverted Axiovert200 Zeiss fluorescence microscope. Astrocytes were considered reactive when they showed hypertrophy and a great number of shorter GFAP-positive processes. The number of positive cells for each marker was divided among each sectiońs DG volumen in order to obtain a cell density (cells/mm^3^). The DG area of each section was estimated delineating de border of the granule cell layer on the same sections where cell numbers was estimated, by using DAPI staining and a 5X objective. Data are presented as mean cell density (cells/mm^3^). All DG areas were measured using Image J software (ImageJ, v. 1.33, NIH, Bethesda, MD, USA, http://rsb.info.nih.gov/ij).

Density of mature granule neurons (Number of cells/mm3) and myc-positive cells was analyzed through the application of a physical-dissector method developed for confocal microscopy (Zeiss LSM710)**,** as described previously [25]. Briefly, the physical dissector was applied to sections stained with DAPI and myc, so all nuclei of mature neurons in the granule cell layer were counted (excluding those nuclei resembling erythrocytes, if any, and the immature cells, easily distinguishable because of its irregular nuclear profiles and highly condensed cromatin). Data are presented as cell density (cells/mm^3^).

### Novelty Suppressed Feeding test

Adult mice (three months) were weighed and food was removed from the cage, although water remained available ad libitum. Twenty-four hours later mice were transferred placed in a novel arena with in the center a pre-weighed quantity of food pellets. Each animal was placed in the corner of the testing area and the latency to chew a food pellet (about 2 g) located in the center of the arena, time spent feeding, and total food consumption were recorded over 10 min. All the experiments were performed between 13:00 and 18:00 h.

### Light/Dark Choice Test

Exploration of the light/dark chamber was measured using the equipment from Med Associates Inc. The mouse is placed for 15 min in a box made of two compartments, one white and lit and the other dark. Two parameters were recorded, the percent of time spent in the dark compartment and the number of transitions between compartments (crossings).

### Statistical analysis

Data were analyzed using two-way ANOVA in dorsal and ventral comparisons between wild-type and transgenic mice. To compare GSK-3 levels and its phosphorylated status in wild-type and GSK3β mice separately, we perform a t-test. All the analyses were performed using SPSS for Windows version 17.0.

## Results

### GSK3β levels in ventral and dorsal DG

**Figure 1:**
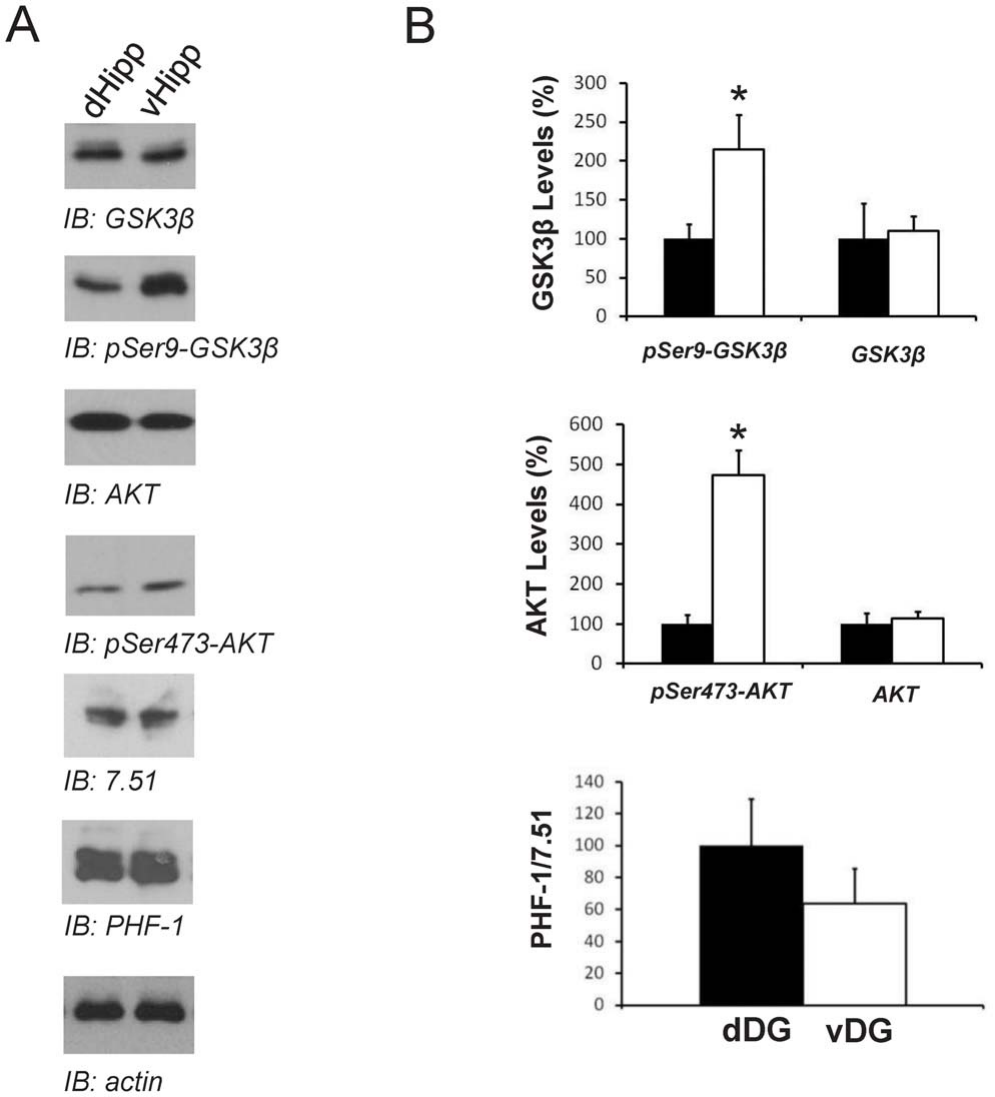
Western blots analysis of GSK3β, AKT and tau proteins in dDG and vDG from wild-type mice. (**A**) Representative western blots showing GSK3β, phosphorylated GSK-3 (pSer9-GSK3β), AKT, phosphorylated AKT (pSer473-AKT), phosphorylated tau (pSer396/404-Tau), tau and actin in homogenates from dorsal and ventral hippocampus of wild-type mice. Hippocampal extracts were prepared from animals aged 2 months. (**B**) Histograms showing the densitometric quantification of samples shown in A. GSK3 and AKT ventral data are expressed in terms of the percentage of signal respect to dorsal levels. Histograms showing tau levels represent phosphorylated tau (PHF-1)/total Tau (7.51). Solid bars, dorsal hippocampus; open bars, ventral hippocampus. *P<0.05 versus dorsal hippocampus.

To examine whether there exist dorso-ventral differences in GSK3β levels in wild-type mice, western blot analysis of dorsal hippocampus and ventral hippocampus homogenates were performed using antibodies that recognize inactive GSK-3β (phosphorylated at serine 9) and with an antibody that recognize GSK3β regardless of its phosphorylation state. Western blot determination of the phosphorylated form of GSK3β revealed an increase in the ratio phosphorylated/unphosphorylated forms in ventral area compared with dorsal area in wild-type mice (Figure 1). The main kinase able to phosphorylate inhibitory GSK3 domains is AKT, thus we analyzed by western blot the hippocampal levels of the active form of AKT that results from phosphorylation on Ser473. Interestingly, and in good agreement with GSK3β findings, AKT is more active ventrally than dorsally. This resulted in a slight (although no statistically significant) decrease in phosphorylated tau, one of the main GSK3 substrates in the nervous system, as demonstrated by western blotting with the PHF-1 antibody. These data strongly suggest that some regional differences exist in the DG with a reduced ventral GSK3β activity compared with dorsal area.

### Transgenic GSK3β is overexpressed in ventral and dorsal DG

**Figure 2:**
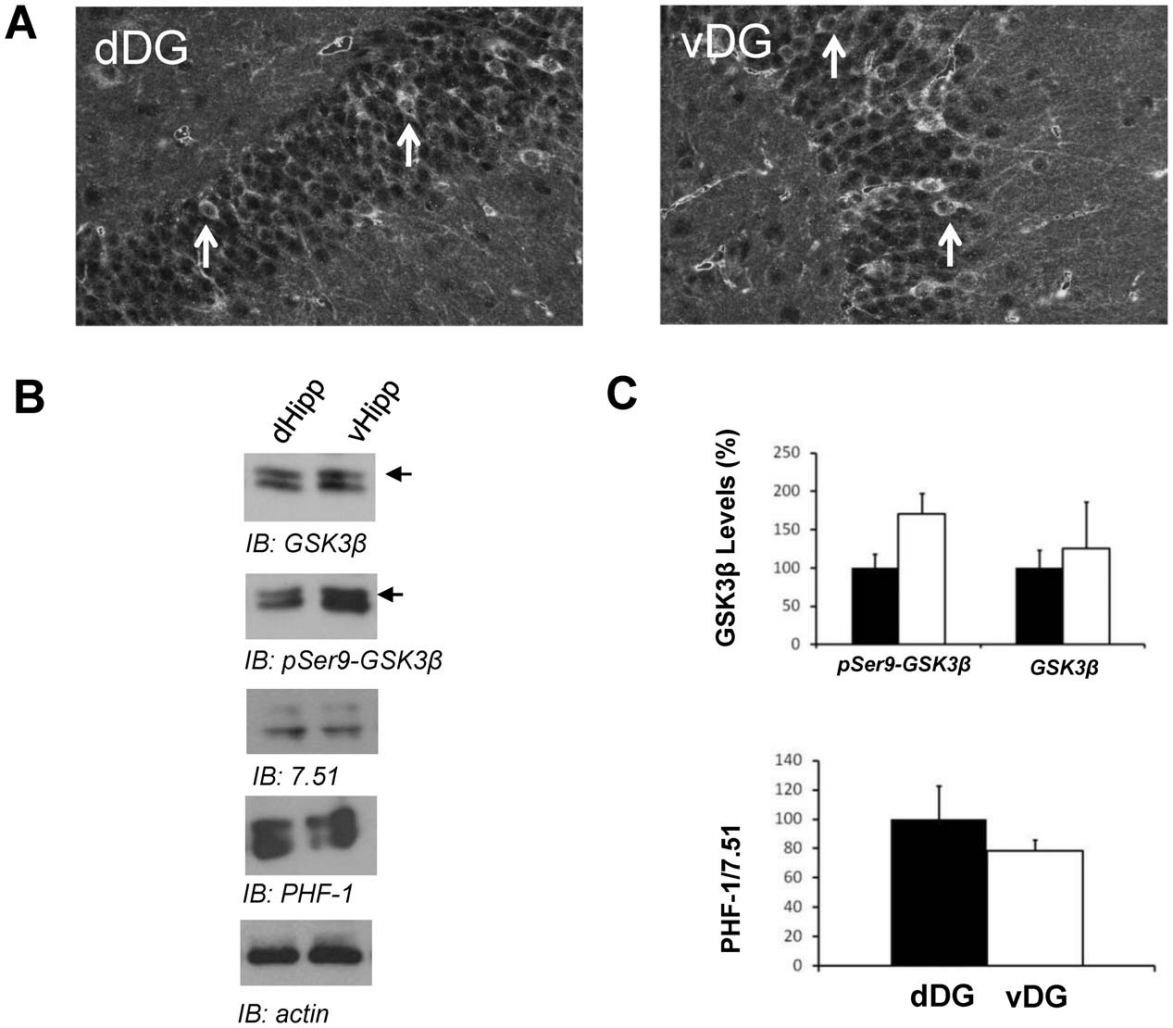
Pattern of transgene expression in GSK3β mice. (**A**) Immunofluorescence in dDG and vDG hippocampal sections of GSK3β mice performed with an antibody against MYC epitope. Arrows shown MYC-positive cells. (**B**) Representative western blots showing GSK3β, phosphorylated GSK-3 (pSer9-GSK3β), phosphorylated tau (pSer396/404-Tau), tau and actin in homogenates from dorsal and ventral hippocampus of GSK3β mice. Hippocampal extracts were prepared from animals aged 2 months. (**C**) Histograms showing the densitometric quantification of samples shown in B. GSK3 data are expressed in terms of the percentage of signal respect to dorsal levels. Histograms showing tau levels represent phosphorylated tau (PHF-1)/total tau (7.51). Solid bars, dorsal hippocampus; open bars, ventral hippocampus (n = 6 dDG; n = 7 vDG).

The tTA transgene is under the control of the calcium/calmodulin kinase IIα promoter allowing expression of transgenes in forebrain neurons. To gain insight into which dentate gyrus area is overexpressing transgenic GSK3β, we performed immunofluorescence with anti-MYC antibody (transgenic GSK3β has a myc epitope at its N-terminal end). Immunofluorescence analysis of sagittal brain sections from GSK3β mice revealed intense staining of the MYC-GSK3β transgene with the anti-myc antibody in granular neurons from dDG and vDG (Fig. 2A). Western-blot analysis was used to confirm that both hippocampal areas show similar transgenic GSK3β levels. Probing protein extracts with anti-GSK3β antibody demonstrated similar levels of transgenic GSK3β (with a slight higher molecular weight due to myc epitope) in both hippocampal areas (Fig. 2B–C). From these experiments we therefore conclude that transgenic GSK3β levels are similar in dDG and vDG. Then, we analyzed Ser9 phosphorylation of total GSK3β. Fig. 2B–C shows, as previously observed in wild-type mice, an slight increase (although no statistically significant) in the levels of inactive GSK3β in ventral area. Tau phosphorylation in dorsal and ventral areas showed a distribution similar to those found in wild-type mice.

### Neurodegeneration is observed in dorsal DG, but not in ventral DG

**Figure 3:**
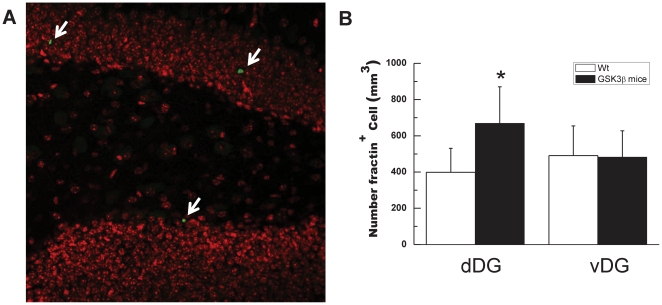
Neural death in GSK3β mice. (**A**) dDG representative image of fractin positive cells (arrows) from GSK3β mice. DAPI staining in red is also shown. (**B**) Quantification of the number of fractin positive neurons in dDG and vDG from wt (n = 9) and GSK3β mice (n = 11). A higher number of fractin positive cells were found in dDG from GSK3β mice compared to wild-type mice. *P<0.001 versus wt animals.

Since overexpression of GSK3β results in apoptotic neuronal death in the dentate gyrus as shown in the initial characterization of GSK3β mice [18], we wondered whether expression of GSK3β in DG of GSK-3β mice might also result in regional apoptosis. To explore in a quantitative manner the incidence of neuronal apoptosis of GSK3β mice, we performed fractin staining (fractin is an actin fragment cleaved by caspase labeling dying cells). In good agreement with previous findings, GSK-3 mice showed an increase in the number of fractin-positive cells in the dDG (an increase of 168±30%. P<0.001, n = 9, Fig. 3), while no a significant difference in the number of fractin-positive cells was detected in the vDG.

**Figure 4:**
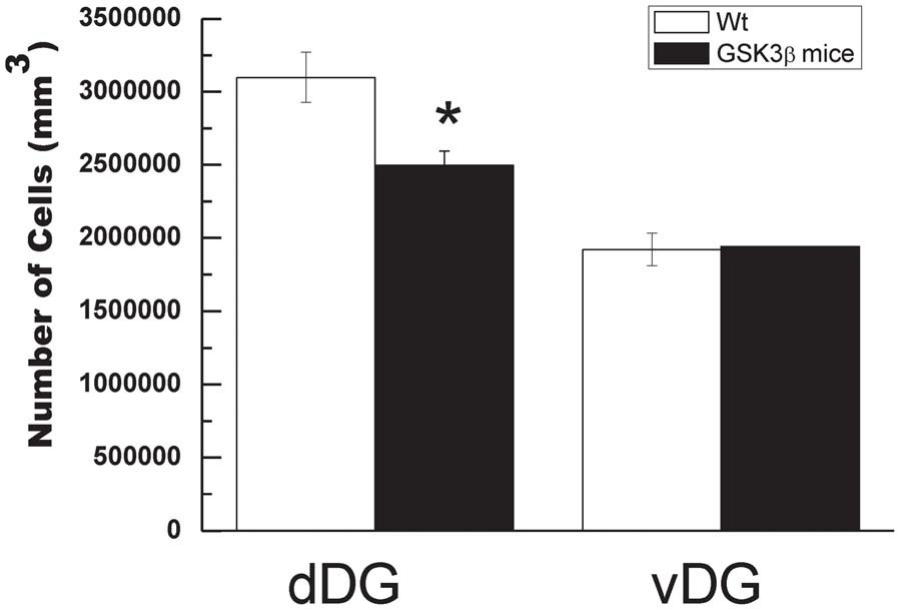
Stimates of total cell number in granule cell layer. Total neurons in 2-mounths old wt and GSK3β mice in dDG and vDG were measured as described in Materials and methods. *P<0.05 versus wt animals.

In view of this previous finding, we decided to test whether dDG increased apoptosis results in a loss of granular neurons through the application of a physical-dissector method in 2-months-old GSK3β mice. As can be seen in Fig. 4 a significant decrease (19.5±0.4%, p<0.001, *n* = 6) in the number of granular neurons was found in 2-months-old GSK3β dDG with respect to their control littermates. We did not find that decrease in vDG.

**Figure 5:**
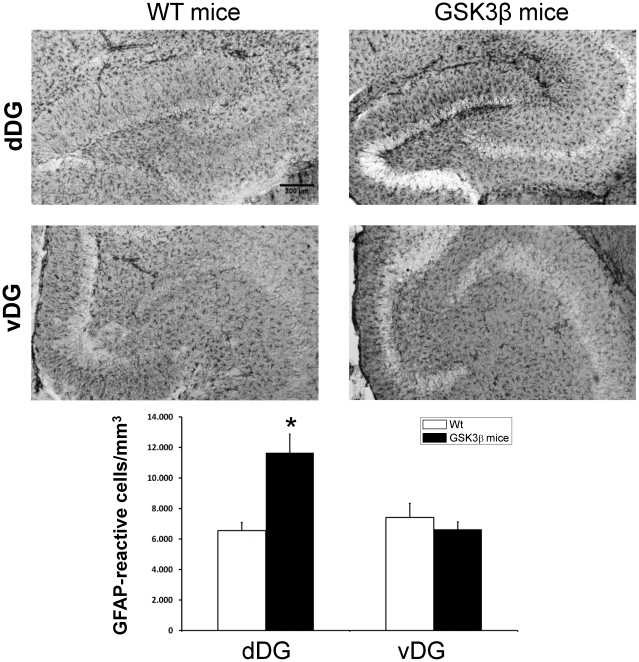
Reactive astrocytosis in dDG compared to vDG in wild-type and GSK3β mice. Immunohistochemistry in hippocampal sections of wild-type (wt) and GSK3β mice performed with an antibodieys against GFAP. Quantification of the number of reactive astrocytes in 2-months-old wild-type (n = 9, wt) or GSK3β mice (n = 11). *P<0.001 versus wt animals.

Reactive astrogliosis often accompanies neuronal loss and serves as a hallmark lesion for neurodegeneration. Thus, we next tested whether the neuronal alterations triggered by overexpression of GSK3β were accompanied by reactive astrocytosis (Figure 5). A remarkable increase in astrogliosis was quite evident in hippocampal dDG (an increase of 177.8±10.5% compared with wild-type mice , p<0.001, n = 11) as shown by immunohistochemistry performed with an antibody raised against GFAP, but not in vDG of GSK3β mice. Together, these results indicate that the neurodegeneration occurs in mice overexpressing GSK3β mainly in hippocampal dDG.

**Figure 6:**
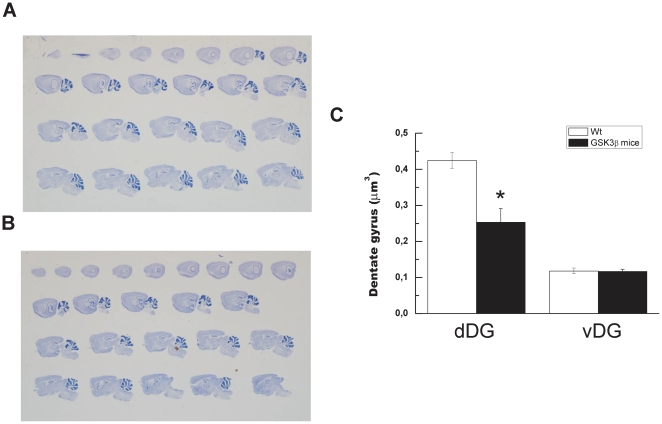
dDG and vDG volume of granule cell layer in old GSK3β mice. Representative sagittal sections from 18-month-old wild-type (**A**) and GSK3β mice (**B**) thionine stained samples used to measured volumetric quantifications. (**C**) Volumetric quantification of the atrophy was determined as described in Material and Methods. *P<0.05 versus wt animals.

We have reported that GSK3β mice present a severe atrophy of total DG, suggesting an important neurodegeneration [7]. To characterize this neurodegeneration, we have previously analyzed thionine-staining in brain sections from two year-old GSK3β mice. Those studies showed a clear reduction in the size of the DG in GSK3β mice when compared to the wt mice. However, we used the entire DG for quantification analysis and a regional study was not performed. When we carried out that kind of analysis a substantial decrease in the dDG of the hippocampus of GSK3β mice was observed. A volumetric analysis of thionine-stained brain sections (Figure 6) revealed that, as compared to wild-type mice, 2-year-old GSK3β mice exhibited an 40.4±7.06% (*P*<0.05, *n* = 3) decrease in dDG volume while vDG was almost unchanged (a decrease of 1.02±2.96%; *P*<0.6, *n* = 3). These data indicate that the neurodegeneration observed in GSK3β mice is mainly due to dorsal area.

### Behavioral consequences of GSK3β overexpression

**Figure 7:**
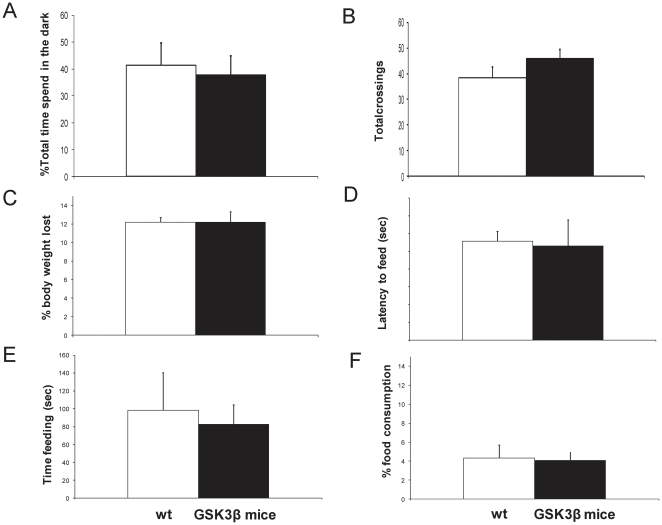
Tests of anxiety-like behavior . Histograms showing the performance of 10-week-old wild-type (wt, white bars, n = 6) or GSK3β mice (black bars, n = 6) in the novelty suppressed feeding test and the white-black box. (A–B) Light/dark box: (**A**) Percent of time spent in the dark compartment (**B**) Number of transitions between the two compartments made by wt and GSK3β mice. (C–F) Novelty supressed feeding test: (**C**) Wild-type and transgenic mice lost a similar body weight during the 24 h of food restriction. Twenty-four hours after food restriction, mice were transferred to the testing room, placed in a novel arena with in the center a pre-weighed quantity of food pellets. Each subject was placed in the corner of the testing area, and the latency to feed (**D**), time spent feeding (**E**), and total food consumption (**F**) were recorded over 10 min. No significant differences were observed.

We have previously demonstrated that an increase in GSK3 activity in the hippocampus is enough to elicit a deficit in spatial learning in the Morris water maze [19]. Learning deficits in GSK3β mice were also evidenced in the object recognition test [20]. These data confirm that hippocampal functions related with dorsal hippocampus are altered in GSK3β mice and those alterations are already present in one-month old animals [20]. To evaluate whether GSK3β overexpression also alter functions related with ventral areas, mice were tested on two tests of anxiety-like behavior: light/dark box and novelty suppressed feeding test (Figure 7). In the light/dark test, GSK3β mice did not show differences respect to wild-type mice in total crossings (T-test, t = 1,395; p = 0.193) or in the amount of time spent in the dark compartment (T-test, t = −0.795; p = 0.445). Similar findings were observed in the novelty suppressed feeding test (Figure 7). Thus, latency to feed in the home cage and amount of food consumed were not different between both groups which suggest again no alterations in motivational factors. Interestingly, no differences in the weight loss during food restriction among transgenic and wild-type mice were detected. These data, with the previously described no differences in the open field test, in terms of total horizontal and vertical activities, time spent in movement, time spent in centre vs. periphery, or stereotypic movements [19] demonstrate that GSK3β mice do not have altered anxiety-like behavior.

## Discussion

The main finding of the current study was to demonstrate that dorsal dentate gyrus is more susceptible to degeneration than ventral area in response to an increase in GSK3β levels, regarding the pattern of transgene expression driven by the CamKIIα promoter, with similar expression in all the hippocampal DG neurons. That conclusion is supported by the following observations: (i) a reduction in the number of granular neurons is observed in dDG of GSK3β mice compared with ventral area; (ii) neuronal death is increased in dDG of GSK3β mice; (iii) overexpression of GSK3β in both DG areas was accompanied by reactive astrocytosis only in the dorsal area; iv) in good agreement, anxiety-like behavior (measured in light/dark box and novelty suppressed feeding tests) is not altered indicating that behavior alterations related with ventral area were not found although a cognitive deficit, related with dorsal areas, is present.

Deregulation of GSK3 has been linked to several prevalent neuropathological conditions. These include bipolar mood disorder [4], schizophrenia [5], diseases like Huntington's disease [6], frontotemporal dementia with parkinsonism linked to chromosome 17 [7] and Alzheimer disease [8]. Therefore, the data reported here on regional consequences of increased GSK3 levels in the DG may have implications in many disease conditions.

Our results show that increased GSK3 levels induces a severe pathology in the hippocampus thus strengthening the hypothesis that GSK3 deregulation may contribute to neurodegeneration. However, important differences seem to exist among neurons. More precisely, some neurons induce an apoptotic program while others seem to survive. Thus, dDG granular neurons undergo apoptotic death (measured as an increase in the number of fractin-positive cells), while vDG neurons do not suffer that processes and survive. The results reported here are indicative of the effects of increased GSK3β levels and, to some extent, they may have implications when considering the potential side effects and the potential therapeutic efficacy of chronic administration of potent and selective GSK-3 inhibitors that are currently under development for treatment of chronic conditions such as Alzheimer's disease and mood disorders [13], [14].

These findings raise the question of how GSK3β overexpression causes dDG degeneration while ventral area seems to be protected. Both regions differ not only in gene expression but also in neuronal connectivity [15]. Taking into account that GSK3β is an enzyme with wide effects on neuronal metabolism, including gene expression [2] neuronal cytoskeleton, plasticity, intracellular transport and neuronal survival, [26] our study suggests that transduction pathways modulating GSK3 are different in both areas.

It is well established that GSK3β activity is inactivated by phosphorylation at Ser9, mainly via AKT, and inhibition of GSK3β is a common event in neuroprotection by different survival factors [2]. We found that GSK3β protein levels were similar in both areas. However, the phosphorylation of AKT at Ser473, which represents its activation, increased in ventral areas and Ser9 phosphorylation of GSK3β was found to be high also in ventral areas. In good correlation, our data seem also to suggest that tau protein phosphorylation, one of the main GSK3 substrates, is slightly less phophorylated in ventral area than in dorsal one. Differences in tau phosphorylation are likely not higher because Ser396/404 epitope is phosphorylated not only by GSK3β but also by other kinases as mitogen-activated protein kinases ERK2, c-Jun N-terminal kinase and P38 [27]. Thus, considering the levels of active AKT and inactive GSK3β, it can be suggested that ventral hippocampus withstands an increase of GSK3 better than dorsal hippocampus. However, further studies are required to specify the mechanisms responsible for those differences as well as the factors making the dDG particularly sensitive to GSK3β mediated apoptosis.

Taking into account our results it is tempting to speculate that some differences may exist among posterior and anterior human DG (which correspond to dorsal and ventral DG in mice [15]) in relation with GSK3. Aberrantly increased GSK3 activity is believed to play a key role in the pathogenesis of mood disorders [2] and in neurodegenerative processes as AD [8]. Focusing in AD, GSK3 has been proposed as the main kinase able to phosphorylate tau aberrantly [28], [29], [30]. Hyperphosphorylated tau does not appear in human dentate gyrus until late stages of Alzheimer's disease [31]. However, mini mental tests demonstrate that cognitive and memory deficits occur in early stages showing that hippocampus is actually altered. Besides, loss of layer II entorhinal neurons occurs in early AD stages [32] suggesting that all these deep changes in the entorhinal input has to have a big impact on it. In fact, although no plaques, tangles or neuronal death appear at early AD stages it has been proposed a partial “disconnection” of the human dentate gyrus in AD [31]. Although actually it is merely speculative to propose that posterior hippocampal human regions are involved in memory and learning processes, while anterior areas are related with emotional and anxiety processes, as it occurs in mouse, functional differences of the anterior versus posterior hippocampus have been reported [33], [34].

In summary, our results demonstrate that dorsal and ventral dentate gyrus do not conduct in a similar way in an animal model overexpressing GSK3β. Thus, the dorsal area is more vulnerable than the ventral one. These results may help to understand partly why in some neurodegenerative process the cognitive and behavior alterations do not come out at the same time. From a more general perspective, our study also suggests that the DG should not be studied as a homogeneous region and studies focused in neurodegeneration should study dorsal and ventral areas.
